# A new concept for the production of ^11^C-labelled radiotracers

**DOI:** 10.1186/s41181-022-00159-y

**Published:** 2022-03-28

**Authors:** Jan Wenz, Felix Arndt, Samuel Samnick

**Affiliations:** Department of Nuclear Medicine, Interdisciplinary PET Center, Universitätsklinikum Würzburg, Oberdürrbacher Strasse 6, 97080 Würzburg, Germany

**Keywords:** Carboxylation, Methylation, Carbon-11, Isotopes, Isotopic labelling, Radiopharmaceuticals, Radiochemistry

## Abstract

**Background:**

The GMP-compliant production of radiopharmaceuticals has been performed using disposable units (cassettes) with a dedicated synthesis module. To expand this “plug ‘n’ synthesize” principle to a broader scope of modules we developed a pressure controlled setup that offers an alternative to the usual stepper motor controlled rotary valves. The new concept was successfully applied to the synthesis of *N*-methyl-[^11^C]choline, *L*-*S*-methyl-[^11^C]methionine and [^11^C]acetate.

**Results:**

The target gas purification of cyclotron produced [^11^C]CO_2_ and subsequent conversion to [^11^C]MeI was carried out on a TRACERlab Fx C Pro module. The labelling reactions were controlled with a TRACERlab Fx FE module. With the presented modular principle we were able to produce *N*-methyl-[^11^C]choline and *L*-*S*-methyl-[^11^C]methionine by loading a reaction loop with neat *N,N'*-dimethylaminoethanol (DMAE) or an ethanol/water mixture of NaOH and *L*-homocysteine (*L*-HC), respectively and a subsequent reaction with [^11^C]MeI. After 18 min *N*-methyl-[^11^C]choline was isolated with 52% decay corrected yield and a radiochemical purity of > 99%. For *L*-*S*-methyl-[^11^C]methionine the total reaction time was 19 min reaction, yielding 25% of pure product (> 97%). The reactor design was used as an exemplary model for the technically challenging [^11^C]acetate synthesis. The disposable unit was filled with 1 mL MeMgCl (0.75 M) in tetrahydrofuran (THF) bevore [^11^C]CO_2_ was passed through. After complete release of [^11^C]CO_2_ the reaction mixture was quenched with water and guided through a series of ion exchangers (H^+^, Ag^+^ and OH^−^). The product was retained on a strong anion exchanger, washed with water and finally extracted with saline. The product mixture was acidified and degassed to separate excess [^11^C]CO_2_ before dispensing. Under these conditions the total reaction time was 18 ± 2 min and pure [^11^C]acetate (n = 10) was isolated with a decay corrected yield of 51 ± 5%.

**Conclusion:**

Herein, we described a novel single use unit for the synthesis of carbon-11 labelled tracers for preclinical and clinical applications of *N*-methyl-[^11^C]choline, *L*-*S*-methyl-[^11^C]methionine and [^11^C]acetate.

**Supplementary Information:**

The online version contains supplementary material available at 10.1186/s41181-022-00159-y.

## Background

Nearly 100 years ago, the production of carbon-11 (^11^C) was reported for the first time (Crane and Lauritsen 1934). The ability to label and metabolically track almost every biologically relevant carbon-containing compound by ^11^C makes this radionuclide an indispensable marker in molecular imaging by positron emission tomography (PET) in modern nuclear medicine.

**Fig. 1:**
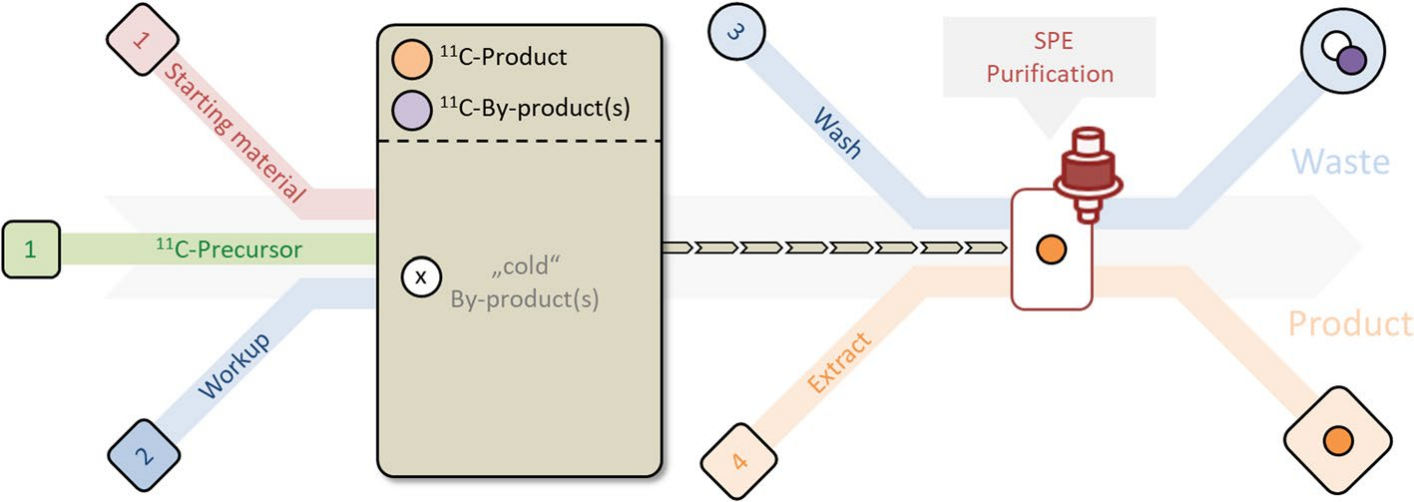
General description of ^11^C-tracer synthesis. 1. Reaction step, 2. Workup and transport of the product mixture to SPE purification cartridge(s), 3. Washing step and 4. Product extraction

A common access to ^11^C-labelled radiopharmaceuticals is the direct activation of [^11^C]CO_2_ with organometallic reagents. Additionally, it can be converted into [^11^C]CH_3_I (Langstrom et al. 1987; Larsen et al. 1997) or [^11^C]CH_3_OTf (Jewett 1992) and then used for radiolabelling. In both cases, the radioactive building block is used in gas form with an inert carrier gas (usually helium). The actual labelling step is performed by bubbling the gas mixture into a reactor, a reaction loop or on a solid matrix. Tracer specific work-up by solid phase extraction (SPE) and cartridge purification has become widely accepted often eliminating the need for time-consuming HPLC separation. A schematic overview is depicted in Fig. 1.

The used materials can have a significant influence on the success or failure of the radio synthesis. To minimize problems, GMP-compliant production of radiopharmaceuticals is preferably performed using very expensive disposable units (cassettes). The synthesis then proceeds according to a “plug ‘n’ synthesize” principle and delivers reliable results. In addition, cross-contamination with other in-house produced pharmaceuticals and handling errors caused by personnel are minimized.

The major clinical benefit of *N*-methyl-[^11^C]choline in PET has been in the evaluation of patients with biochemically recurrent prostate cancer (Hara et al. 1998; Scattoni et al. 2007; Reske et al. 2008; Rinnab et al. 2008; Mitchell et al. 2012), and currently, for localization of parathyroid adenoma (Orevi et al. 2014; Liu et al. 2020; Noltes et al. 2021). *L*-*S*-methyl-[^11^C]methionine is a ^11^C-labelled analogue of the essential amino acid methionine. It enters several metabolic pathways and has been used in various applications such as imaging brain tumours (Sato et al. 1992), hyperparathyroidism (Sundin et al. 1996; Rubello et al. 2006), neck and head tumours (Lindholm et al. 1993). Currently it is used for the evaluation of multiple myeloma including the staging, the prognostication and the assessment of therapy’s response (Lapa et al. 2017; Morales-Lozano et al. 2020; Lückerath et al. 2015). The synthesis of both *N*-methyl-[^11^C]choline and *L*-*S*-methyl-[^11^C]methionine are quite robust and they are produced routinely in many institutions with a cyclotron.

[^11^C]Acetate has been used as imaging agent for studying myocardial oxidative metabolism (Henes et al. 1989; Grassi et al. 2012; Nesterov et al. 2015) and regional myocardial blood flow (Gropler et al. 1991) as well as for prostate cancers (Oyama et al. 2002).

**Fig. 2:**
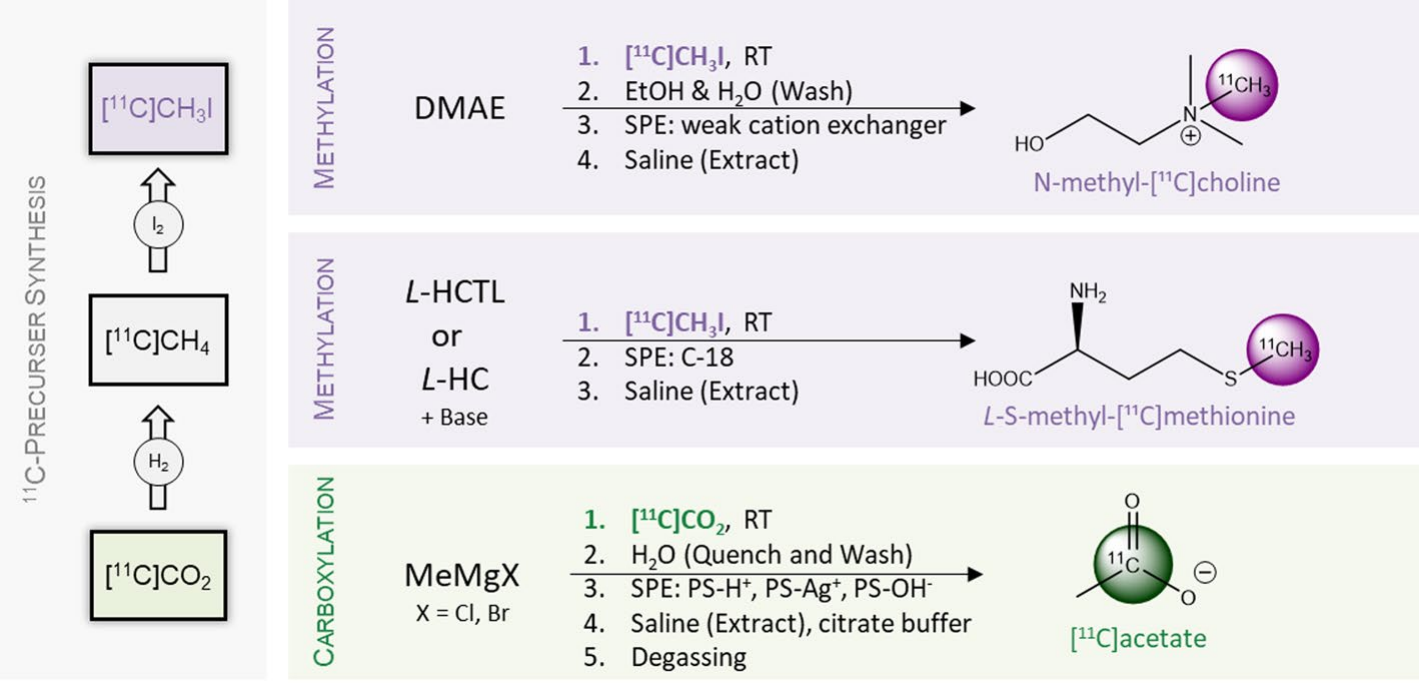
Radiochemistry overview. Left: Gas chemistry for the preparation of ^11^C-precursors. Purple: Methylation of *N*,*N′*-dimethylaminoethanol (DMAE) and conversion to *N*-methyl-[^11^C]choline and methylation of L-homocystein (HC) or L-homocysteinthiolactone (HCTL) to produce L-*S*-methyl-[^11^C]methionine. Green: Carboxylation reaction for the production of [^11^C]acetate with methyl magnesium chloride/bromide

The most common method for the production of [^11^C]CO_2_ is the irradiation of a ^14^N_2_ target with protons following the reaction [^14^N(p,α)^11^C]. The unavoidable presence of [^16^O]oxygen (^16^O_2_) traces in the target gas leads to the formation of undesired [^13^N]nitrogen during the irradiation, that is present in form of nitrogen oxides (NO_x_) and has to be removed before further conversions (Ache and Wolf 1966). Modern synthesis units use molecular sieves for this purpose, as they can be regenerated and used over a long period of time with low maintenance. This process starts by binding [^11^C]CO_2_ selectively onto the molecular sieve. Subsequent purging with an inert carrier gas removes the unwanted by-products. At high temperatures (> 250 °C) pure [^11^C]CO_2_ is released and can either be used directly for the synthesis (CO_2_ Bypass) or be converted to [^11^C]methyl-iodide. In the so-called "gas phase” conversion it is reduced with hydrogen at 360 °C to [^11^C]methane on a heterogeneous nickel catalyst and then reacted with iodine at 720 °C to produce [^11^C]CH_3_I (Larsen et al. 1997) (Fig. 2 left). The individual syntheses for *N*-methyl-[^11^C]choline (Pascali et al. 2000; Jinming et al. 2006; Kuznetsova et al. 2003), *L*-*S*-methyl-[^11^C]methionine (Lodi et al. 2008) and [^11^C]acetate (Berridge et al. 1995; Kruijer et al. 1995; Roeda et al. 2002; Soloviev and Tamburella 2006; Kang Se et al. 2016; Maurer et al. 2019; Mitterhauser et al. 2004) have been optimised in the last decades and are reviewed in detail (Oleksiy et al. 2013; Gomzina et al. 2015; Dahl et al. 2017). A general overview is shown in Fig. 2.

While the requirements toward materials and equipment for ^11^C methylation reactions are undemanding, as no aggressive solvents or sensitive reagents are required. The conditions for carboxylation reactions, however, require the use of organometallic compounds. These are stable in organic solvents such as tetrahydrofuran or diethyl ether which are not compatible with many polymers used in regular disposable cassettes due to possible corrosion of the material. Furthermore, organometallic reagents are sensitive to air and moisture, which also leads to difficulties in handling.

Despite the important applications, there is currently no cassette-like system that can fulfil all these requirements. This prompted us to develop a novel flexible disposable setup for the synthesis of tracers according to the synthesis principle shown in Fig. 1.

## Methods

### General

#### Radionuclide production

[^11^C]CO_2_ was prepared by bombardment of ^14^N_2_ target gas containing 1% O_2_ with protons (10–60 µA, 16.5 MeV) using a PETtrace 860 cyclotron (GE Healthcare).

#### ^11^C-precursor production

**Fig. 3:**
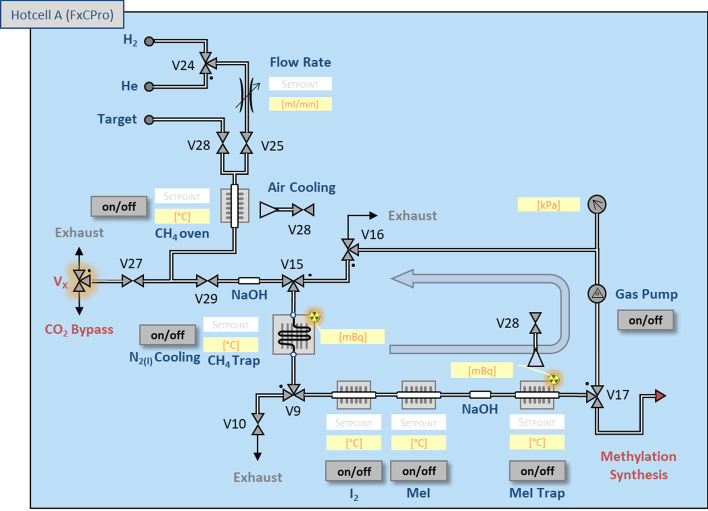
Configuration of the TRACERlab FX C Pro module. The installation of V_x_ valve (orange) allows a simple CO_2_ bypass

For the production of ^11^C-labelled compounds from [^11^C]CO_2_ a TRACERlab FX C Pro (GE Healthcare) system was used as synthesis module. To use purified [^11^C]CO_2_ directly valve (V_x_) was installed (CO_2_ bypass, see Fig. 3). No further modifications to the module were made (for details see Additional file 1: Tables S1, S2).

#### General modifications to the TRACERlab FX FE Pro

**Fig. 4:**
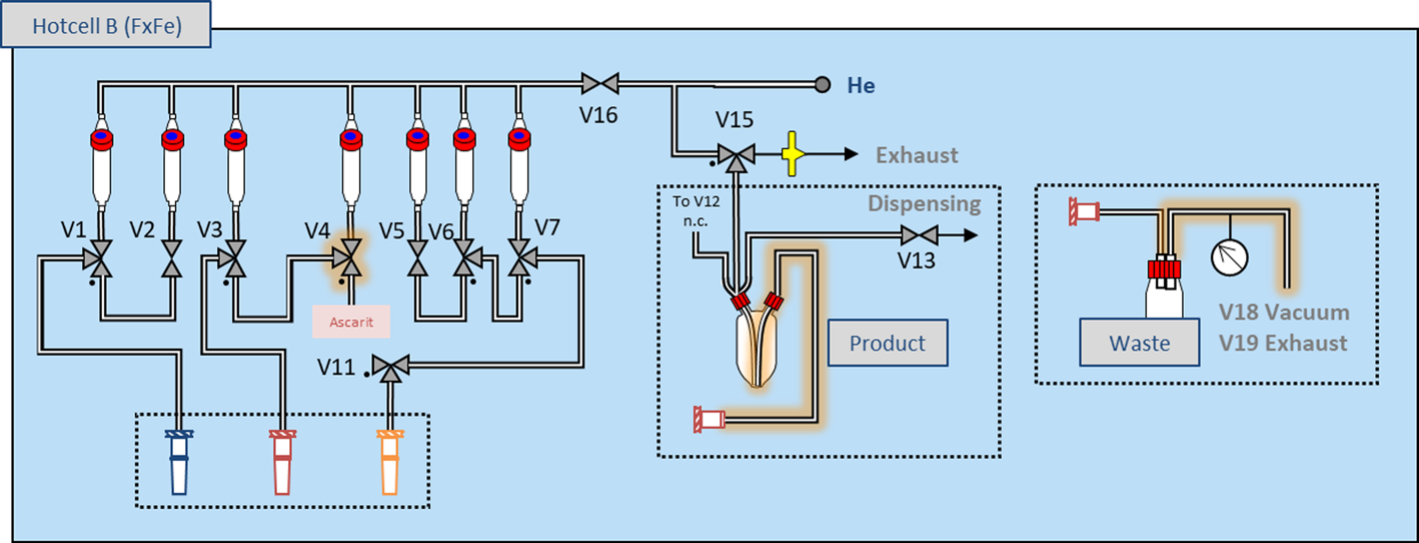
Configuration of the TRACERlab FX FE module. The modifications compared to the standard setup are highlighted in orange

The synthesis module was cleaned and dried before each synthesis and used as a control unit for the disposable setup. The 2-way valve V4 was replaced by a 3-way valve and connected to an ascarit®(II) trap. This allowed pressure exhaust during the carboxylation reaction and trapping of unreacted [^11^C]CO_2_. The V18 and V19 lines which are usually connected to the module reactor, were connected to the waste vessel instead. The product vessel was filled via the usually closed second neck with a tube or cannula reaching to the bottom of the vial (See Fig. 4). The reactors activity detector was dismounted and placed close to the disposable unit to monitor the activity profile during the reaction.

### Disposable materials

For the assembly of the disposable synthesis units the following materials were used: Check valves (AMT Medica, Germany, Ref: 0079), luer T Shape Adapter, FFM, PP (Cole Parmer Instruments, Germany, Ref: OU-45508-85), luer T Shape Adapter, FMM, PP (Cole Parmer Instruments, Germany, Ref: OU-45508-75), loop: PE Lectrocath extension 1.5 × 2.5 mm, length 150 cm (Vygon, Germany, Ref: 1159.10), reactor: 10 mL luer lock PP Syringe Body without plunger (DB Plastic, Ref: 300912) sealed with a rubber septum or crimped with a butylrubber plug with cap R 20B-L (CS chromatographie, Germany, Ref: 300242). Cannulas: Sterican® 1.20 × 40 mm, orange (B. Braun), Sterican® 0.6 × 60 mm, blue BL/LB), Optional: PE Lectrocath extension lines 1.5 × 2.5 mm, length 30–150 cm available (Vygon, Germany). Ion exchange cartridge for *N*-methyl-[^11^C]choline workup: Weak cation exchanger, Sep-Pak Accell Plus CM Plus Short cartridge (Waters, Ref: WAT020550), 360 mg sorbent per cartridge, 37–55 µm, was used without conditioning. SPE cartridge for *L*-*S*-methyl-[^11^C]methionine production: Sep-Pak C18 Plus Short Cartridge (Waters, Ref: WAT020515), 360 mg Sorbent per Cartridge, 55–105 µm, was used without conditioning. Ion exchange cartridge for [^11^C]acetate: Strong cation exchangers: PS-H^+^ (230 mg, Macherey–Nagel, Düren, Germany Ref: 731861) and 3 × PS-Ag^+^ (3 × 240 mg, Macherey–Nagel, Düren, Germany Ref: 731865), strong anion exchanger: PS-OH^–^ (200 mg, Macherey–Nagel, Düren, Germany, Ref: 731860), were pre-conditioned with 10 mL of ethanol and 10 mL of water and blown dry with 10 mL air.

## Reagents

2-Dimethylamino-ethanol (Sigma Aldrich, Ref: 471453), *L*-homocysteine (*L*-HC) (Sigma Aldrich, Ref: 69453), 0.9% Saline (USP, B. Braun, Ref: 2737756) and Ethanol (Supelco, ACS, ISO, Reag. Ph Eur., Merck, Ref: 1.00983) were used without prior purification. Water (Milli-Q) was obtained from a Milli-Q Ultrapure Water system. NaOH_aq._ 50% (c = 19.05 M, Supelco, Merck, Ref: 158793) was diluted with Milli-Q water to the appropriate concentration. Sodium citrate buffer solution (139 mM, pH = 4.7) was prepared from citric acid (Sigma Aldrich, Ref: 251275) according to literature (Maurer et al. 2019). 3 M MeMgCl in THF (Sigma Aldrich, Ref: 189901) and THF (ExtraDry, stored over molsieve, stabilized AcroSeal™, Acros, Ref: 10292182) were used without prior purification to prepare a 0.75 M MeMgCl stock solution using standard Schlenk technique. This solution was stored under argon in a 25 mL Schlenk flask (Glasgerätebau Ochs, Germany, Ref: 143514) and is stable for at least 3 months at room temperature.

### Radiolabelling

#### *N*-Methyl-[^11^C]choline

The solvent reservoirs of the TRACERlab FX FE module were prepared as following: Vial 1: 4 mL 0.9% Saline (Extract), vial 2: Empty, vial 3: Empty, vial 4: Empty, vial 5: Empty, vial 6: 5 mL water (Wash), vial 7: 5 mL ethanol (Wash), product vessel: Empty, reaction loop: 100 µl DMAE.

**Fig. 5:**
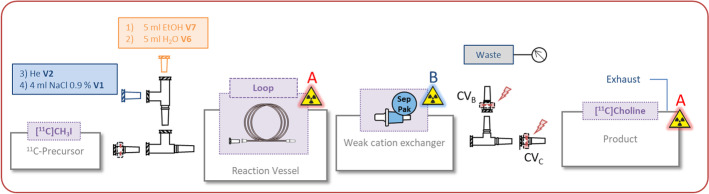
Setup for *N*-methyl-[^11^C]choline

Process description: The disposable unit was assembled as described in Fig. 5 and the loop was filled with DMAE. The SPE cartridge was placed in front of the activity detector of the reactor (B) and the reaction loop was positioned close to the product detector (A). The solvent reservoirs were connected and the synthesis program was started. Monitoring and semi-automatic control was performed by using the pressure and activity detectors (for details see Additional file 1: Table S3).

Quality control: The radiochemical purity was determined by HPLC (Dionex, Ion Pac CS12A, l = 25 cm, d = 4 mm, cation exchange resin), mobile phase 0.02 M methanesulfonic acid, flow 1.0 mL/min, R_t_ (*N*-methyl-[^11^C]Choline) = 7.5 min.

#### *L*-*S*-Methyl-[^11^C]methionine

The solvent reservoirs of the TRACERlab FX FE were were prepared as following: Vial 1: 4 mL 0.9% Saline (Extract), vial 2: Empty, vial 3: Empty, vial 4: Empty, vial 5: Empty, vial 6: Empty, vial 7: Empty, product vessel: Empty, reaction Loop: 1.5 mg *L*-homocysteine (*L*-HC), 190 µmol NaOH in 210 µl H_2_O and 200 µl ethanol were mixed in an eppendorf vial and filled into the loop.

**Fig. 6:**
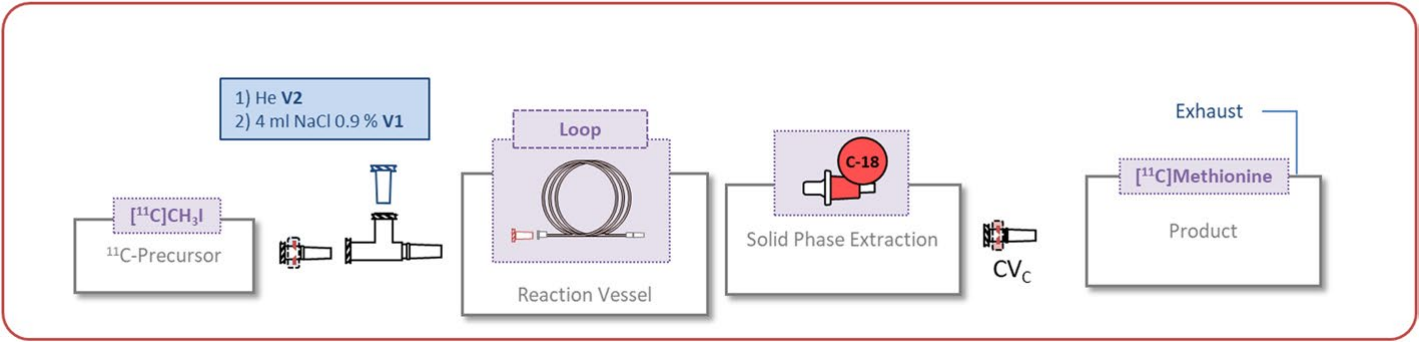
Setup for L-*S*-methyl-[^11^C]methionine

Process description: The disposable unit was assembled as described in Fig. 6 and the loop was filled with the precursor solution. The solvent reservoirs were connected and the synthesis program was started (for details see Additional file 1: Table S4).

Quality control: The radiochemical purity was determined by HPLC (Phenomenex, Gemini® 5 µm, C18, 110 Å, l = 15 cm, d = 4.6 mm, C-18 Resin), mobile phase = 0.05 M NaH_2_PO_4_ / 2% ethanol, pH = 6.2 – 6.3, flow = 0.75 mL/min, R_t_ (*S*-methyl-[^11^C]Methionine) = 3.4 min.

#### [^11^C]Acetate

**Fig. 7:**
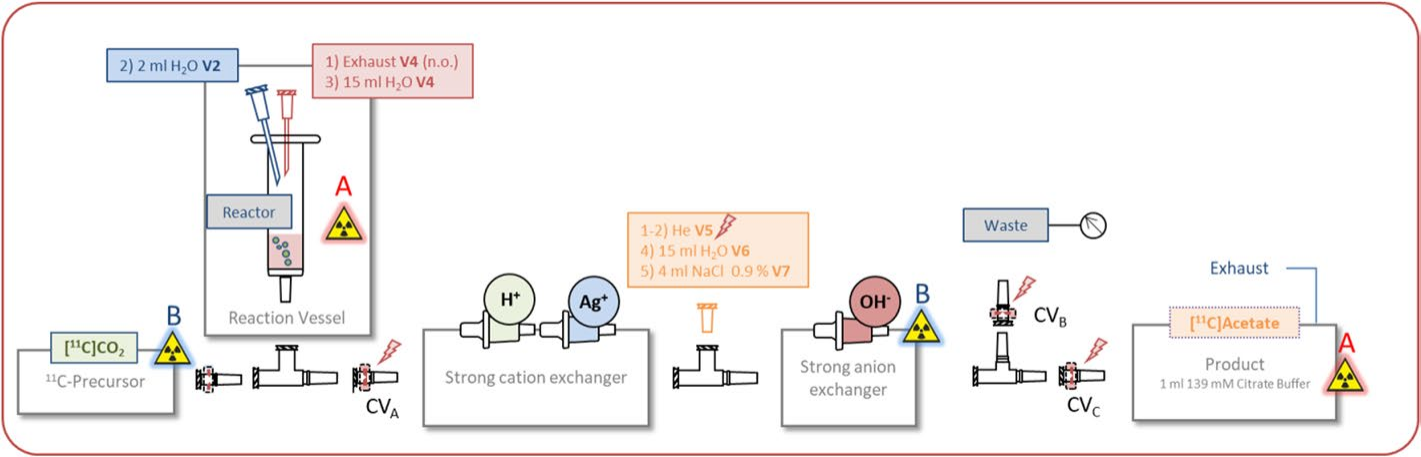
Setup for [^11^C]acetate

The disposable unit was assembled as described in Fig. 7 and the solvent reservoirs of the TRACERlab FX FE module were prepared as following: Vial 1: Empty, vial 2: 2 mL water (to quench the reaction), vial 3: Empty, vial 4: 15 mL Water (for transport and washing), vial 5: Empty. Vial 6: 15 mL Water (Washing), Vial 7: 4 mL 0.9% Saline (Product extraction from anion exchange cartridge), product vessel: 1 mL citrate buffer (for removal of [^11^C]CO_3_^2−^).

Process description: The ion exchangers were pre-conditioned and the parts were assembled as described in Fig. 7. The solvent reservoirs were connected and the synthesis program was started. A helium overpressure was generated via V5 and CV_A_ was closed. The reactor was purged with dry argon for 5 min via the "normally CO_2_" input. Then the MeMgCl solution was carefully added to the reactor via the septum. Afterwards the argon line was exchanged for the [^11^C]CO_2_ line. The anion exchange cartridge was placed in front of the reactor activity detector (B) and the reaction vessel was positioned close to the product detector (A) (for a detailed description see Additional file 1: Table S5).

Quality control: The radiochemical purity was determined by HPLC (CarboPac PA10, Dionex, Thermofisher Scientific, l = 25 cm, d = 4 mm, particle size 10 µm), mobile phase: 0.1 M NaOH, flow 1.0 mL/min, R_t_ ([^11^C]Acetate) = 8–10 min, TLC (NaOH impregnated silica plates), mobile phase: methanol 100%, R_f_ ([^11^C]Acetate) = 0.8–0.9 in accordance with (Berridge et al. 1995; Maurer et al. 2019).

## Results

The loop configuration for the synthesis of *N*-methyl-[^11^C]choline and *L*-*S*-methyl-[^11^C]methionine was chosen. Neat *N*,*N′* dimethylaminoethanol (DMAE) or an ethanol/water mixture of NaOH and *L*-homocysteine (*L*-HC) were loaded into a polyethylene loop and reacted with [^11^C]MeI for 180 s. The target gas purification and conversion to [^11^C]MeI was carried out on a TRACERlab FxCPro module. The labelling reactions were controlled with a TRACERlab FxFE module. As a proof of concept A_EOB_ = 3700 MBq of [^11^C]CO_2_ were used as a starting material and 898 MBq (decay corrected 52%) of [^11^C]choline were isolated after 22 min. For the synthesis of [^11^C]methionine A_EOB_ = 25 GBq of [^11^C]CO_2_ were used to produce 3 GBq (decay corrected 25%) *L*-*S*-methyl-[^11^C]methionine after 23 min (19 min reaction + 3 min filling). The results are similar to our routine production and the yield and purity are in the range of the syntheses described in the literature and have not been further optimised.

Since clogging during hydrolysis of the MeMgCl THF solution is a major problem in [^11^C]acetate synthesis, a reactor design was chosen to prevents this issue. The reaction was optimised by irradiation of the gas target for 2 min with 10 µA beam current yielding A_EOB_([^11^C]CO_2_) = 2000 MBq. After 18 ± 2 min, we were able to isolate the desired product in 52 ± 5% (n = 9) yield. And to simulate clinically relevant conditions, a higher starting activity of [^11^C]CO_2_ (A_EOB_ = 25 GBq) by irradiating the target for 5 min with 60 µA beam current was used as well. The exemplarily diagrams shown in Table 1 were taken from this experiment. The total troduction time was 17 min and 6.6 GBq (decay corrected 51%) of the product could be isolated.

**Table 1:** Short description and in-process control of a representative [^11^C]acetate synthesis using a TRACERlab FX FE module


Step	Duration	Description
Pre-synthesis	Approximately 30 min before EOB	Check valve A (CV_A_) was closed by helium overpressure from V5. The reactor was filled with MeMgCl THF solution and purged with argon. Finally the [^11^C]CO_2_ line was connected
Radiolabelling	EOB + 8 min	The [^11^C]CO_2_ line was positioned close to the product detector (B) so that a temporary increase in activity was observed. After the reaction an activity maximum was measured in the reaction vessel
Quench and wash	2–4 min^a^	The reaction mixture was quenched (Vial 2) and washed with water (Vial 4). The check valves A and B were opened by closing V5 and applying a vacuum to the waste vessel. An increase in activity on the anion exchange cartridge was observed. A pressure increase in the waste vessel indicates that the washing step has been completed
Wash anion exchanger	60 s	To ensure that all by-products were separated, the anion exchanger cartridge was washed again with water from V6. A pressure increase in the waste vessel indicates that the washing step has been completed
Helium purge	30 s	Further purging with helium closes check valve B (CV_B_)
Product extraction and degassing	90 s	By releasing the overpressure from the product vial, the check valve C (CV_C_) was opened and the product was extracted from the anion exchanger with saline. The product was transferred into the citrate buffer solution and the by.product [^11^C]carbonate was removed by helium bubbling
Filling	180 s	Filling

^a^This step can take from 2 to about 4 min, as the reaction mixture must pass through five ion exchangers

In order to estimate the activity distribution, all the components of the synthesis apparatus were dismounted and analysed after the reaction. The decay corrected activity was 8% for the exhaust ascarite®(II) trap of the reactor, 4% for the PS-H^+^ cartridge, 7% for the 3 Ag^+^ cartridges and 3% for the anion exchanger. 14% of the total activity was measured in the waste vessel. The exhaust line of the product vessel was also connected to an ascarite®(II) trap, the received activity is in direct correlation to the carbonate content (8%) of the saline extraction solution. Eight percent of the total activity was not found. We suspect that these traces remained on the remaining parts.

Quality control was performed according to literature and was in accordance with monograph of Pham Eu. for [^11^C]acetate and *L*-*S*-methyl-[^11^C]methionine injection solutions. The results are summarized in Table 2.

**Table 2:** Results for the ^11^C-tracer production

^11^C-Tracer	*N*-Methyl-[^11^C]Choline	*L*-*S*-Methyl-[^11^C]Methionine	[^11^C]Acetate
Reaction type	Methylation		Carboxylation
Time^[a]^	18 min	19 min	18 ± 2 min
Isolated Yield	900 MBq^[b]^	3000 MBq^[c]^	6600 MBq^[c]^
Yield^[d]^ on [^11^C]CH_3_I^[e]^	85%	42%	-
Yield^[d]^ on [^11^C]CO_2_	52%	25%	52 ± 5%, (n = 10)

^[a]^Total reaction time including radioprecursor synthesis: [^11^C]CH_3_I (14 min) or [^11^C]CO_2_ Bypass (8 min)

^[b]^Irradiation parameters 20 µA, 2 min ([^11^C]CO_2_ A(EOB)_cal_ = 3.7 GBq)

^[c]^60 µA, 5 min ([^11^C[CO_2_ A(EOB)_cal_ = 25 GBq)

^[d]^Decay corrected at EOB

^[e]^Gas phase conversion to ^11^CH_3_I 60%

## Discussion

**Fig. 8:**
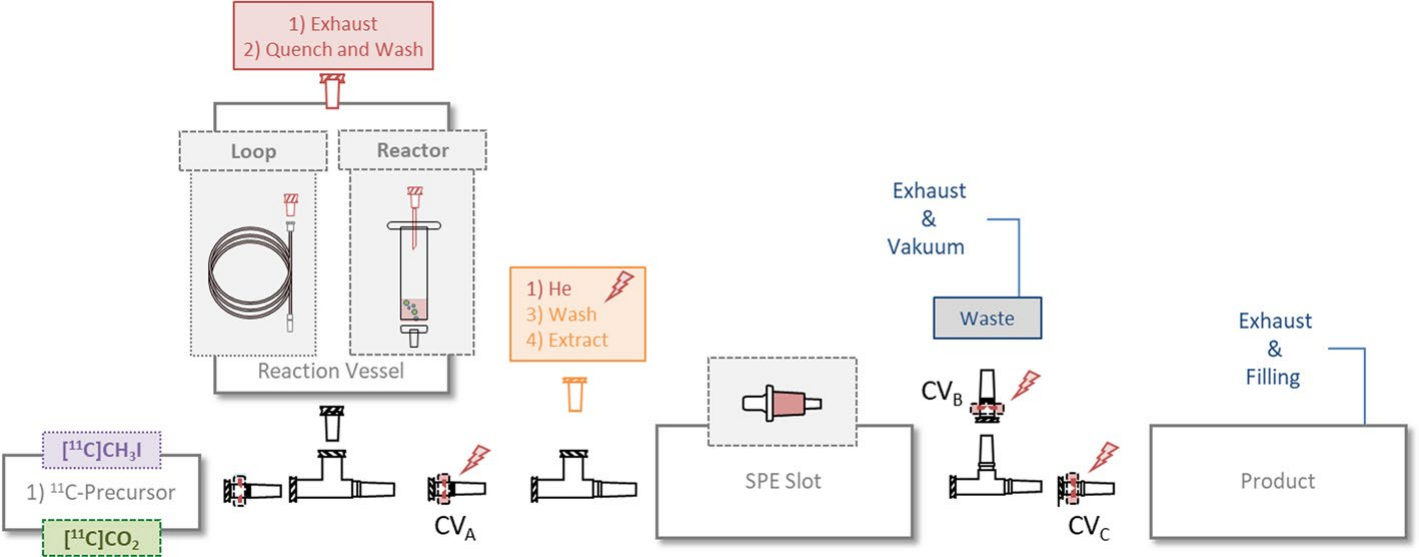
General setup for radiotracer synthesis. Left to right: Gas inlet check valve for the ^11^C-precursor. T-Adapter with reaction vessel (loop or reactor). Check valve A and T-adapter for SPE cleaning. Separation valve with CV_B_ and CV_C_ for product isolation. Valve configuration: Reaction (all CVs closed), wash (CV_C_ closed) and extraction (CV_B_ closed). Opening and closing of the valves is controlled by gas pressure (here helium marked red)

The main objective was to use disposable, commercially available materials without complicated modifications. A summarized general setup is shown Fig. 8. The individual reaction paths were controlled externally by pressure regulation- and the special feature is the use of check valves or even 3-way valves as control units.

The basic principle of this assembly consists of pluggable elements that allow convenient customisation. T-shaped luer adapters, which are available in various designs serve as the framework. A “loop” or a “reactor” setup is possible in both cases. For the latter, a disposable syringe body with septum is used. Luer check valves are used to attach the desired SPE cartridges for cleaning. Additionally, a T-shaped valve is connected at the end to separate wash and extraction solution. Solvent and gas supply were controlled by the synthesis module. A typical reaction proceeds as following: The check valves CV_A_, CV_B_ and CV_C_ are closed by a gas overpressure (via the orange adapter) and the gaseous radio-precursor is then passed through a check valve into the apparatus. The gas flow is automatically directed into the reactor or reaction loop where the chemical reaction takes place. Pressure equalisation is permitted through the connected exhaust line. The gas supply is then terminated and the waste vessel evacuated. This opens CV_A_ and CV_B_ and the reaction mixture can be guided through the SPE cartridge and be washed. Once all washing steps are completed, purging with Helium continues until the generated overpressure closes CV_B_ again. By opening the exhaust valve at the product vessel CV_C_ is opened. The product can then be extracted, sterile filtered and dispensed.

[^11^C]Choline and [^11^C]methionine are produced on a regular basis in our clinic. The total yield for methylation reactions is largely dependent on the efficient production of [^11^C]methyliodide. This process produced about 60% decay corrected [^11^C]MeI with respect to the produced [^11^CO]_2_ in the period of our study. Since the general reaction conditions were not changed, both the reaction times and the yields obtained are within the range of the syntheses described in literature (Oleksiy et al. 2013; Gomzina et al. 2015; Dahl et al. 2017).

Due to the high sensitivity to water, the use of disposable products is especially desirable for Grignard reagents. The first attempts for the [^11^C]acetate synthesis were performed with a loop setup were as well. Despite a very precise work, we were not able to achieve a stable and reliable reaction. Fluctuating yields, high carbonate content in the reaction mixture and occasionally clogging of the reaction loop was observed. Therefore, further experiments were carried out in a disposable syringe as a reactor.

With the selected helium gradient (Additional file 1: Table S1) for the delivery of the purified [^11^C]CO_2_ from the molecular sieve an efficient trapping in 1 mL THF (even without Grignard) was achieved. After 180 s of gas injection, less than 10% of the total activity was monitored on the exhaust ascarite trap.

Reliable and constant results were obtained using similar conditions reported by Kang Se et al*.* (2016). Methyl magnesium chloride (1 mL, 0.75 M) in tetrahydrofuran was used as an alkylation reagent. To guarantee a constant concentration a stock solution was used for all experiments. Under these conditions, about 60% [^11^C]acetate was formed. The remaining activity was split among the known by-products [^11^C]acetone, [^11^C]*tert*-butanole and [^11^C]carbonate. A lower Grignard concentration led to a higher formation of [^11^C]carbonate, an increase resulted in an elevated formation of [^11^C]acetone and [^11^C]*tert*-butanole.

Product trapping on an anion exchange resin can only be effective when all the chloride (0.75 mmol) is removed from the reaction mixture (Kruijer et al. 1995). In order to verify the latter, we added an aqueous silver nitrate solution (1%) to the reaction mixture after the cation exchanger procedure. The absence of a colourless precipitate indicated complete chloride removal. Thus, the ion exchangers were adjusted to the corresponding amount of Grignard reagent in “cold” pre-tests.

As the reaction mixture needed to pass through five ion exchanger cartridges, the waste vessel was evacuated during the washing process. The uncharged by-products [^11^C]acetone and [^11^C]*tert*-butanole were separated completely within an acceptable period of time. After extraction with saline, the mixture of anionic components [^11^C]acetate and [^11^C]carbonate was transferred into aqueous citrate buffer solution (1 mL, 139 mM). The resulting solution had a pH between 5 and 6 and by helium bubbling for 90 s [^11^C]carbonate was efficiently removed as [^11^C]CO_2_ (Maurer et al. 2019). In summary, we were able to produce [^11^C]acetate within 17 ± 2 min and decay corrected yield of 51 ± 5% pure (n = 10).

## Conclusion

The used materials are sterile, affordable and single-use products. Furthermore, the key materials are available in inert materials, such as polyethylene (PE) or polypropylene (PP) which is essential for the use of sensitive chemicals and organic solvents. As a large variety of modules allow pressure/vacuum control, this design can easily be adapted for specific application. This concept may not only help in the establishment of new ^11^C-tracers for research and routine clinical applications but also to improve the synthesis of established ^11^C-labelled PET-tracers, especially, when no cassette based modules are available or reactive chemicals have to be used. We are convinced that this setup system can also be adopted to other tracers. The extension of this concept to routine tracers was part of our current research. The first experience shows that the new unit can successfully be applied to the synthesis of [^11^C]choline, [^11^C]methionine and [^11^C]acetate with good radiochemical yields in less than 20 min synthesis time.

## Supplementary Information

**Additional file 1.** Additional information for N-methyl-[^11^C]choline, L-S-methyl-[^11^C]methionine and [^11^C]acetate synthesis.

## Data Availability

The datasets used and/or analysed during the current study are available from the corresponding author upon reason-able request.

## Abbreviations

GMP: Good manufacturing practice
DMAE: *N,N′* Dimethylaminoethanol
d.c.: Decay corrected
THF: Tetrahydrofuran
PET: Positron emission tomography
SPE: Solid phase extraction
HPLC: High performance liquid chromatography
*L*-HC: *L*-Homocystein
*L*-HCTL: *L*-Homocysteinethiolactone
EOB: End of bombardment
PE: Polyethylene
PP: Polypropylene
