# Supplementary material for: A new concept for the production of 11C-labelled radiotracers
Source: EJNMMI Radiopharm Chem. 2022 Mar 28;7:6. doi: 10.1186/s41181-022-00159-y (PMC8960519; doi:10.1186/s41181-022-00159-y)
Supplement: Supplementary file 1 — Additional file 1. Additional information for N-methyl-[11C]choline, L-S-methyl-[11C]methionine and [11C]acetate synthesis. [file 41181_2022_159_MOESM1_ESM.docx]

**Supporting Information**

**[^11^C]CO_2_:** Before each synthesis, the CH_4_ oven was conditioned with helium at 350 °C for 20 min and then cooled to <35 °C before end of bombardment (EOB). Cyclotron produced [^11^C]CO_2_ is trapped in a 4 Å MS at room temperature a released by heating the oven up to 380 °C. To achieve a complete activity transfer, a helium gradient was chosen. The total duration of this procedure was about 8 minutes.

Supplementary table 1 Description, time list and in-process control of [^11^C]CO_2_ trapping and release.

| **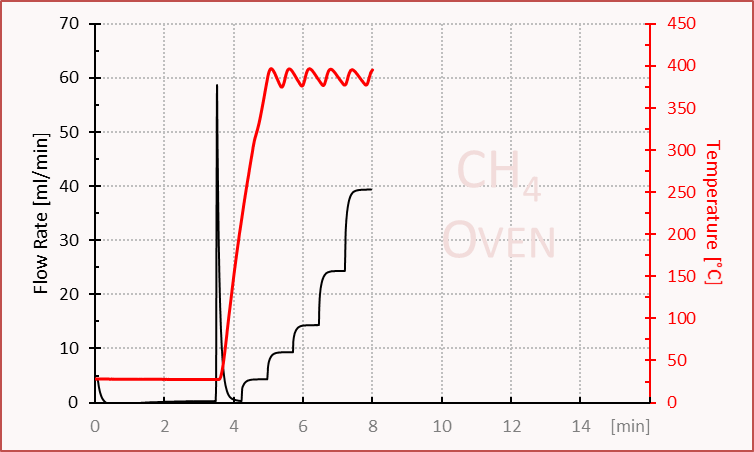** | | | | | |
| --- | --- | --- | --- | --- | --- |
| **Time** |  | **Device** |  | **Value** | **Comment** |
| 0 | Set | Power | = | on | V.210416 |
| 2 | Set | Flow rate Set Point | = | 5 |  |
| 4 | Set | V28 | = | Open | Cooling on |
| 6 | Set | V24 | = | a (down) |  |
| 8 | Set | V25 | = | Open |  |
| 10 | Set | V27 | = | Open |  |
| 12 | Set | Process Control | = | Show Message and wait | System ready to receive activity |
| t1+2 | Set | V25 | = | Close |  |
| t1+3 | Set | V26 | = | Open | C11 activity release |
| t1+5 | Set | V27 | = | Open |  |
| t1+10 | Set | Select counter | = | 3B on, 3a Off |  |
| t1+11 | Get | Activity Product |  |  |  |
| t1+4’0 | Set | V25 | = | Open |  |
| t1+4’0 | Set | V26 | = | Close | C11 Activity stop |
| t1+4’1 | Set | V28 | = | Close | Cooling off |
| t1+4’2 | Set | V27 | = | Open |  |
| t1+4’3 | Set | Temp. Set Point CH4 | = | 300 |  |
| t1+4’3 | Wait | Temp. Reg. Status CH4 | = | Temp OK |  |
| t2+0 | Set | VX | = | b (up) |  |
| t2+2 | Set | Temp. Set Point CH4 | = | 380 |  |
| t2+3 | Wait | Temp. Reg. Status CH4 | = | Temp. OK |  |
| t3+1 | Set | Flow Rate Set point | = | 10 |  |
| t3+45 | Set | Flow Rate Set Point | = | 15 |  |
| t3+1’30 | Set | Flow Rate Set Point | = | 25 |  |
| t3+2’15 | Set | Flow Rate Set Point | = | 40 |  |
| t3+3’0 | Set | V27 | = | Close |  |
| t3+3’0 | Set | V28 | = | Open |  |
| t3+3’1 | Set | V25 | = | Reset |  |
| t3+3’1 | Set | V25 | = | Close |  |

**[^11^C]CH_3_I:** The general synthesis process of labelling with [^11^C]methyliodide is described as following (see service manual TRACERlab FX C Pro). Preparation: Before each synthesis, the CH_4_ oven was conditioned with hydrogen at 350 °C for 20 min and then cooled to <45 °C before end of bombardment (EOB). Cyclotron produced [^11^C]CO_2_ was trapped on 4 Å molecular sieve and online converted by Ni-catalyst (Shimalite-Ni) support, under continuous addition of hydrogen at 360 °C. Both, formed H_2_O and unconverted [^11^C]CO_2_ are held back by an ascarite-trap (immobilized NaOH). The produced [^11^C]methane was trapped on a Carbosphere resin (60-80 mesh) under N_2_ liq. cooling at -75 °C for further purification and concentration. Afterwards the purified [^11^C]CH_4_ was reacted with iodine at 720 °C to form [^11^C]methyliodide in a gas circulating process system. Formed HI was removed by a separate ascarite trap. [^11^C]Methyliodide was separated from this circulation process by a trap containing Porapak Q (50-80 mesh) as adsorbents at room temperature. Unconverted [^11^C]CH_4_ returns into the circulation process for reaction with iodine. The collected [^11^C]methyliodide was released into a moderate helium flow off the adsorbents (190 °C) and used for further labelling. The total time of this procedure was about 15 minutes.

Supplementary table 2 Description, time list and in-process control of [^11^C]CH_3_I Production and release.

| **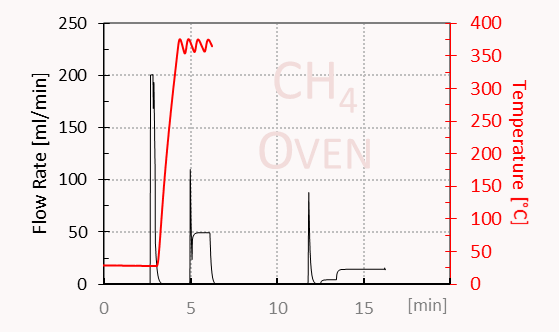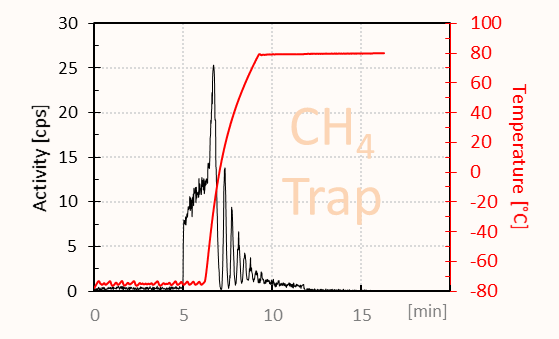**  **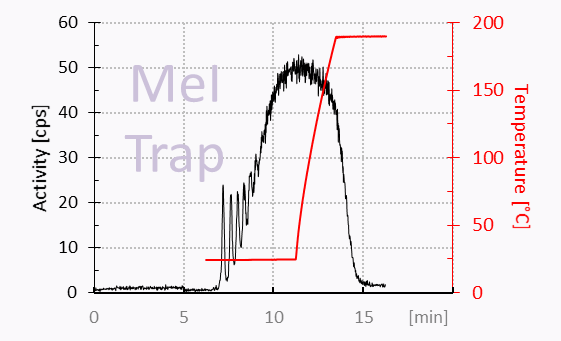** | | | | | |
| --- | --- | --- | --- | --- | --- |
| **Time** |  | **Device** |  | **Value** | **Comment** |
| 0 | Set | Power | = | on | MeI for Choline Modified |
| 0.4 | Set | Temp. Reg. Switch MeI | = | on |  |
| 0.6 | Set | Flow Rate Set Point | = | 20 |  |
| 1 | Set | V25 | = | open |  |
| 5 | Set | V28 | = | open |  |
| 6 | Wait | Temp. CH4 | <= | 45 |  |
| t1+0 | Set | Process Control | = | Show message and wait | Ready for CH4 trap cooling |
| t2+0 | Set | Temp. Set Point CH4 Trap | = | -75 |  |
| t2+1 | Wait | Temp. Reg. Status CH4 Trap | = | Temp. OK |  |
| t3+0 | Set | Process Control | = | Show message and wait | System ready to receive activity |
| t4+0 | Set | Temp. Set Point I2 | = | 100 |  |
| t4+1 | Get | Flow Rate Set Point | = | 0 |  |
| t4+2 | Set | V25 | = | Close |  |
| t4+2.5 | Set | V26 | = | Open | C11 activity release |
| t4+3 | Set | V27 | = | Open |  |
| t4+4’9 | Set | V26 | = | Close | C11 activity stop |
| t4+4’10 | Set | V24 | = | B (up) | H2 flow |
| t4+4’11 | Wait | Flow Rate Set Point | = | 200 |  |
| t4+4’12 | Set | V25 | = | Open |  |
| t4+4’13 | Set | Flow Rate | >= | 45 |  |
| t5+5 | Set | V27 | = | Close |  |
| t5+10 | Set | V28 | = | Close |  |
| t5+15 | Set | V25 | = | Close |  |
| t5+16 | Set | V24 | = | A (down) | Switch to He |
| t5+17 | Set | Flow Rate Set Point | = | 50 |  |
| t5+18 | Set | Temp. Set Point CH4 | = | 360 |  |
| t5+19 | Wait | Temp. Reg. Status CH4 | = | Temp. OK |  |
| t6+40 | Set | V10 Exhaust | = | Open |  |
| t6+41 | Set | V15 CH4 Trap Input | = | B (left) |  |
| t6+42 | Set | V29 | = | Open |  |
| t6+43 | Set | V25 | = | Open | Purge and transport with He |
| t6+1’40 | Set | V09 | = | B(right) |  |
| t6+1’41 | Set | V16 Exhaust | = | B(up) |  |
| t6+1’5 | Set | V25 | = | Close |  |
| t6+1’51 | Set | V29 | = | Close |  |
| t6+1’53 | Set | V15 CH4 Trap Input | = | A (right) |  |
| t6+1’54 | Set | V16 Exhaust | = | A(down) |  |
| t6+1’55 | Set | Gas Pump | = | On |  |
| t6+1’56 | Set | V16 Exhaust | = | B (up) |  |
| t6+1’56 | Set | Temp. Set Point CH4 Trap | = | 80 |  |
| t6+1’56 | Set | Temp. Reg. Switch CH4 | = | Off |  |
| t6+1'58 | Set | Select Heater | = | 3B on, 3 A off |  |
| t6+1'58 | Set | V16 Exhaust | = | A (down) |  |
| t6+4'20 | Set | Process Control | = | Show message and wait | Press o.k. when activity on MeI-Trap constant |
| t7+0 | Set | Temp. Set Point MeI Trap | = | 190 |  |
| t7+30 | Set | Gas Pump | = | Off | Circulation |
| t7+31.9 | Set | V15 CH4 Trap Input | = | B (left) |  |
| t7+32.9 | Set | V29 | = | Open |  |
| t7+33.9 | Set | V25 | = | Open |  |
| t7+34.9 | Set | V16 Exhaust | = | B (up) |  |
| t7+36 | Set | Flow Rate Set Point | = | 5 |  |
| t7+40 | Set | V17 | = | B (down) |  |
| t7+41 | Set | V16 Exhaust | = | A (down) |  |
| t7+42 | Set | V08 | = | Open |  |
| t7+43 | Wait | Temp. MeI Trap | >= | 185 |  |
| t8+1 | Set | Flow Rate Set Point | = | 15 |  |
| t8+1'10 | Set | Process Control | = | Show message and wait | Press Okay when MeI has been transferred |

Supplementary table 3 Description, time list and in-process control of N-methyl-[^11^C]choline Synthesis using a TRACERlab FX FE module.

| **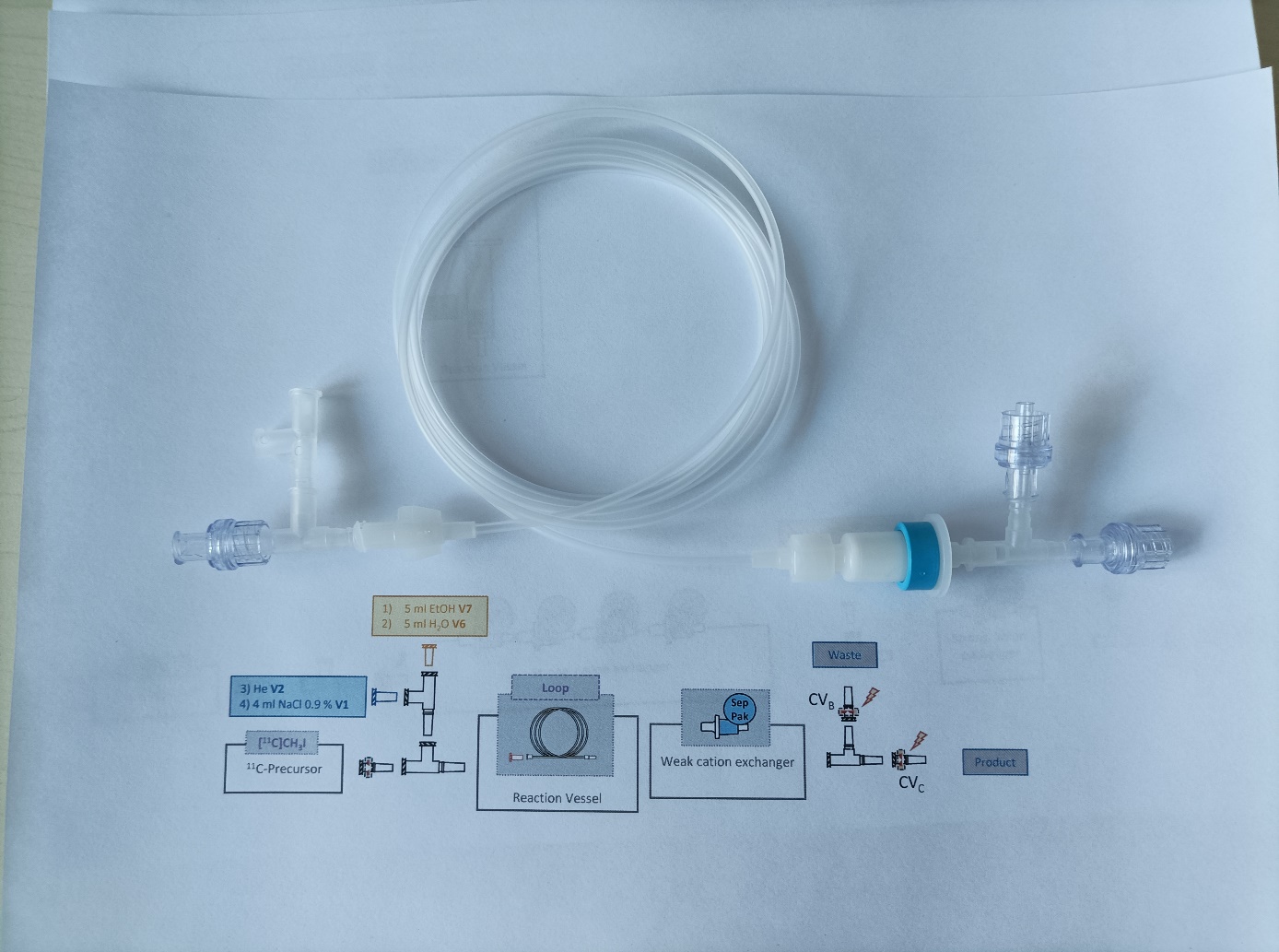**  **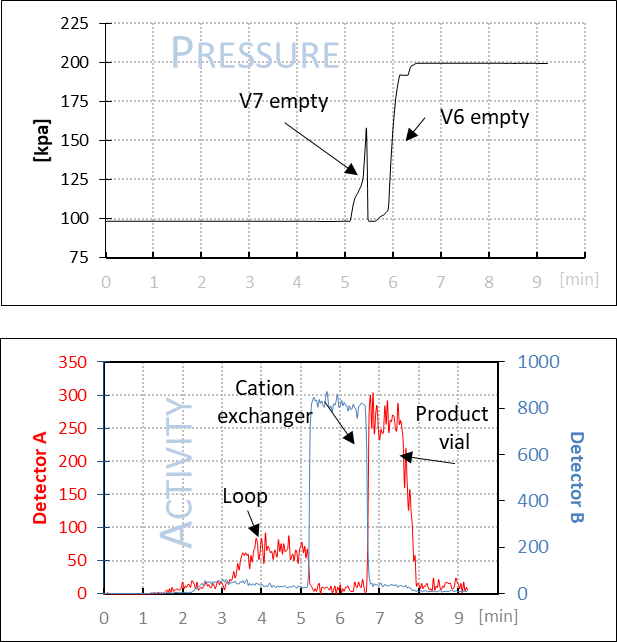** | | | | |
| --- | --- | --- | --- | --- |
| **Step** | **Time** | **Device** | **Value** | **Comment** |
| Radiolabelling | 0 | V11 | b (right) | Check valve B (CV_B_) was open and CV_C_ was closed. After complete release of [^11^C]CH_3_I the operator started the workup. |
|  | 0 | V15 | b (left) |  |
|  | 0 | V16 | Open |  |
|  | 0 | V19 | Open |  |
|  | 5 | Activity Product |  |  |
|  | 5 | Activity Reactor |  |  |
|  | 10 | Process Control | **Show message and wait** |  |
| Ethanol Wash | t1+5 | V19 | Close | To remove unreacted starting material, the system was washed with ethanol. At the same time, an increase of activity on the cartridge was observed. A pressure increase in the waste container indicates that the washing step has been completed. |
|  | t1+7 | V07 Vial7 | b (up) |  |
|  | t1+20 | Process Control | **Show message and wait** |  |
|  | t2+1 | V07 Vial7 | a (down) |  |
|  | t2+1 | V19 | open |  |
|  | t2+10 | V19 | close |  |
| Water Wash | t2+11 | V06 Vial6 | b (up) | To remove the ethanol residues from the cartridge, the system was then washed with water. The activity on the cartridge remains constant during this process. A pressure increase indicated the end of this step. |
|  | t2+35 | Process Control | **Show message and wait** |  |
| He Purge | t3+1 | V06 Vial6 | a (down) | Further purging with helium closes check valve B (CV_B_) |
|  | t3+10 | V19 | Close |  |
|  | t3+11 | V02 Vial2 | Open |  |
|  | t3+20 | Process Control | **Show message and wait** |  |
| Product Extraction | t4+1 | V02 Vial2 | Close | By releasing the pressure from the product vial, the check valve C was opened and the product was extracted from the cation exchanger with saline. |
|  | t4+2 | V15 | a (right) |  |
|  | t4+3 | V01 Vial1 | Open |  |
|  | t4+1'0 | Process Control | **Show message and wait** |  |
| Filling | t5+1 | V15 | b (left) | Filling |
|  | t5+2 | V01 Vial1 | Close |  |
|  | t5+3 | V13 | Open |  |
|  | t5+1'0 | Process Control | **Show message and wait** |  |
| End | t6+1 | Process Control | Reset |  |

Supplementary table 4 Description and time list of L-S-methyl-[^11^C]methionine synthesis using the TRACERlab FX FE module.

| **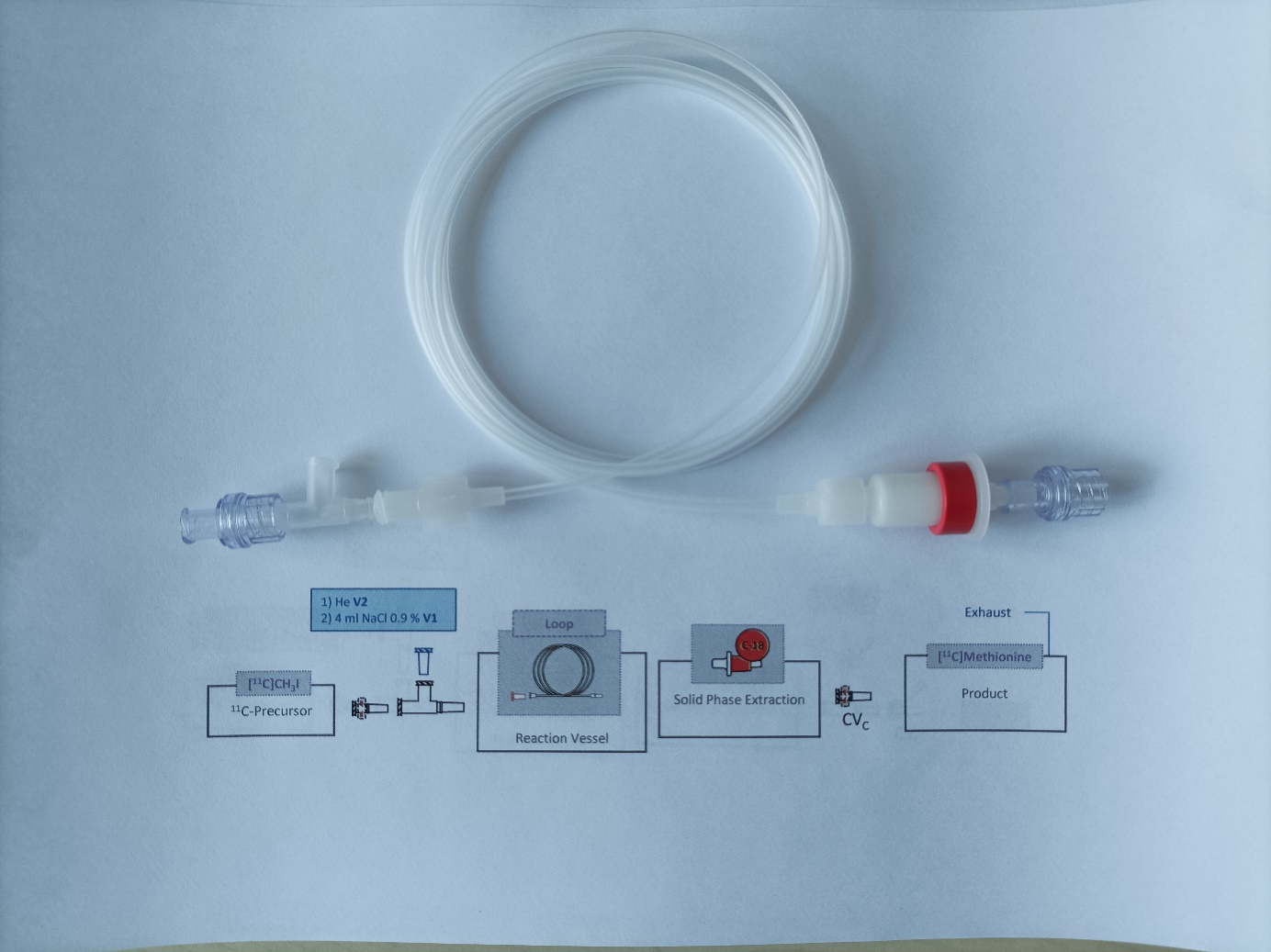** | | | | | |
| --- | --- | --- | --- | --- | --- |
| **Step** | **Time** | **Device** | **Value** | **Comment** | |
| Radiolabelling | 15 | Process Control | **Show message and wait** | After complete release of [^11^C]CH_3_I the operator started the workup. | |
| He Purge | t1+5 | V02 Vial2 | open | After the reaction was completed, the aperture was purged with helium to remove traces of unreacted methyliodide. | |
|  | t1+1’30 | V02 Vial2 | close |  |  |
| Product Extraction | t1+1’31 | V01 Vial1 | b (up) | Product extraction | |
|  | t1+2’30 | Process Control | **Show message and wait** |  |  |
| Filling | t2+1 | V15 | b (left) | Filling | |
|  | t2+2 | V01 Vial1 | Close |  |  |
|  | t2+3 | V13 | Open |  |  |
|  | t2+1'0 | Process Control | **Show message and wait** |  |  |
| End | t3+1 | Process Control | Reset |  |  |

Supplementary table 5 Description, time list and in process control of a representative [^11^C]acetate synthesis using the TRACERlab FX FE module.

| **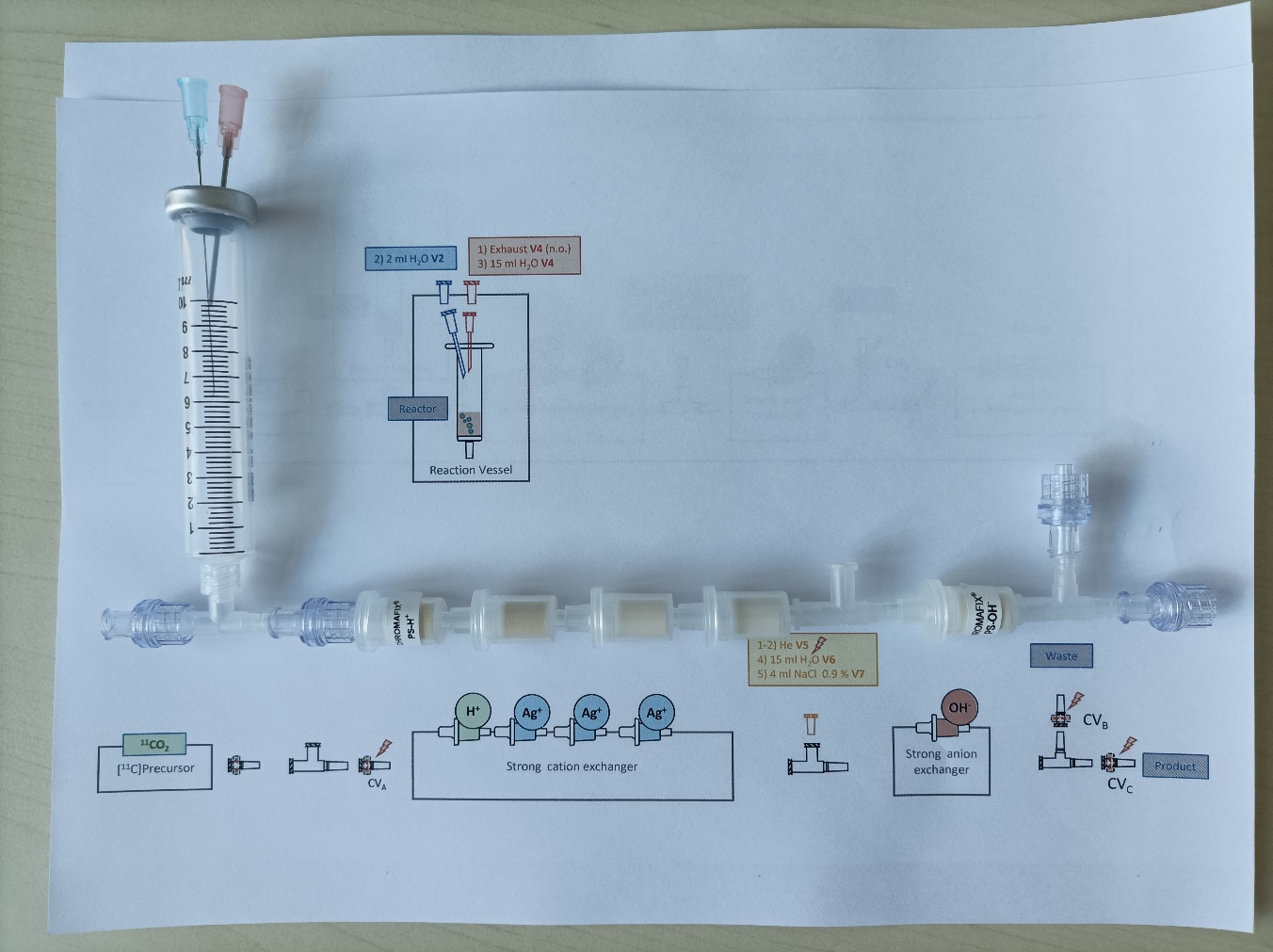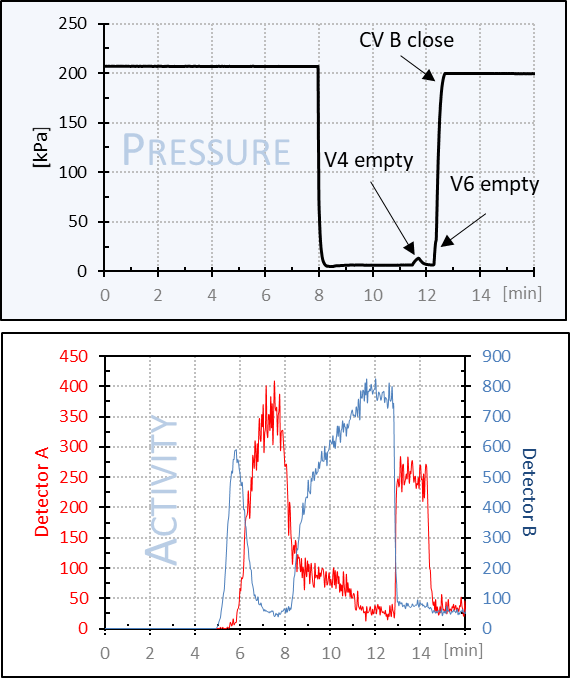** | | | | |
| --- | --- | --- | --- | --- |
| **Step** | **Time** | **Device** | **Value** | **Comment** |
| Pre-synthesis | 0 | V05 Vial5 | open | Check valve A (CV_A_) was closed by helium overpressure from V5. The reactor was filled with Grignard reagent. The latter was purged with argon and the [^11^C]CO_2_ line was connected. |
|  | 0 | V11 | b (right) |  |
|  | 0 | V15 | b (left) |  |
|  | 0 | V16 | Open |  |
|  | 10 | Process Control | Show message and wait |  |
| Radiolabelling | t1+5 | Activity Product |  | The [^11^C]CO_2_ line was close to the product detector so temporary an increase in activity was observed. After the reaction, the highest activity was measured in the reaction vessel. |
|  | t1+6 | Activity Reactor |  |  |
|  | t1+10 | Process Control | Show message and wait |  |
| Quench and Wash | t2+10 | V02 Vial2 | Open | The reaction mixture was quenched and washed with water. The check valves A and B were opened by applying a vacuum. An increase in activity on the anion exchange cartridge was observed. A pressure increase in the waste vessel indicates that the washing step has been completed. |
|  | t2+20 | V02 Vial2 | Close |  |
|  | t2+20 | Power | On |  |
|  | t2+20.1 | V05 Vial5 | Close |  |
|  | t2+25 | V18 | Open |  |
|  | t2+25.1 | V04 Vial4 | Open |  |
|  | t2+30 | Process Control | Show message and wait |  |
| Wash wash anion exchanger | t3+5 | V04 Vial4 | Close | To ensure that all by-products were separated, the anion exchanger cartridge was washed again with water. A pressure increase in the waste vessel indicates that the washing step has been completed. |
|  | t3+10 | Vial06 Vial6 | b (up) |  |
|  | t3+20 | Process Control | Show message and wait |  |
| Helium Purge | t4+5 | V06 Vial6 | a(down) | Further purging with helium closes check valve B (CV_B_). |
|  | t4+5.1 | V05 Vial5 | Open |  |
|  | t4+5.2 | V18 | Close |  |
|  | t4+5.3 | Power | Off |  |
|  | t4+10 | Process Control | Show message and wait |  |
| Product extraction and degassing | t5+1 | V05 Vial5 | Close | By releasing the pressure from the product vial, the check valve C (CV_C_) was opened and the product was extracted by ion exchange with saline. The product is transferred into the citrate buffer solution and [^11^C]carbonate was removed by helium bubbling. |
|  | t5+5 | V05 Vial5 | Close |  |
|  | t5+5.1 | V15 | a (right) |  |
|  | t5+5.2 | V07 Vial7 | b (up) |  |
|  | t5+1’30 | Process Control | Show message and wait |  |
| Filling | t6+5 | V07 Vial7 | a (down) | Filling |
|  | t6+10 | Process Control | Show message and wait |  |
|  | t7+5 | V15 | b (left) |  |
|  | t7+5.1 | V13 | Open |  |
|  | t7+30 | Process Control | Show message and wait |  |
| End | t8+1 | Process Control | Reset |  |
